# Digital tomosynthesis spot view in architectural distortions: outcomes in management and radiation dose

**DOI:** 10.1007/s11547-022-01570-w

**Published:** 2022-12-19

**Authors:** Valeria Fiaschetti, Nicolo’ Ubaldi, Smeralda De Fazio, Aurora Ricci, Federico Maspes, Elsa Cossu

**Affiliations:** 1 Department of Biomedicine and Prevention, Tor Vergata University, Rome, Italy; 2 Department of Medical Surgical Sciences and Translational Medicine, Sapienza - University of Rome, Radiology Unit – Sant’Andrea Hospital, 1035 Via Di Grottarossa, 00189 Rome, Italy; 3 European Hospital, Rome, Italy; 4 UOC of Diagnostic Imaging, Policlinico Tor Vergata (PTV) University, Rome, Italy

**Keywords:** Architectural distortion, Digital breast tomosynthesis, Digital breast tomosynthesis spot compression view, Negative predictive value of digital breast tomosynthesis, Average glandular dose

## Abstract

**Purpose:**

To evaluate if digital breast tomosynthesis spot compression view (DBT-SCV) could be an additional projection to confirm or deny architectural distortions (ADs) detected by digital breast tomosynthesis (DBT) while assessing the average glandular radiation dose.

**Methods:**

This is a retrospective cohort study enrolling 8864 DBT exams, of which only cases detecting primary AD and with BI-RADS 2–5 score were considered. Seventy-one AD cases examined with DBT-SCV, US and MRI were evaluated for correlation in terms of BI-RADS score; variables among exams were assessed for inter-relationships.

**Results:**

Of all ADs identified at DBT, biopsy yielded malignancy in only 38%. PPV in identifying malignancy of ADs was higher for DBT-SCV than DBT (*p* < 0.05); the NPV of DBT-SCV was 94%. The difference between DBT and DBT-SCV in the detection of benign ADs was statistically significant (*p* < 0.05). AD without US or MRI confirmation was less likely to represent malignancy (*p* < 0.05). In detecting malignant cases of ADs, both DBT and DBT-SCV were strongly correlated with US and RM (Kappa > 0.90). In identifying benign cases of ADs, DBT-SCV was poorly/moderately correlated with US and RM (Kappa 0.25 and 0.66); DBT was negatively correlated with US and MRI.

**Conclusion:**

DBT-SCV could be useful to better characterize AD firstly identified by DBT, keeping dose levels within the reference limits. If AD is detected by DBT without an US or MRI correlate, that is not confirmed by DBT-SCV, a “wait and see” approach can be applied to reduce unnecessary biopsy.

## Introduction

Architectural distortion (AD) is the third most commonly missed mammographic presentation of non-palpable breast cancer, accounting for almost 6% of the abnormalities detected by screening mammography [1]. Compared to microcalcifications or a clinically palpable mass, architectural distortion can be more difficult to identify, due to its often-blurred morphology, especially within dense breasts. Under the umbrella of AD, defined as “alteration of normal breast architecture in the absence of a visible mass” by BI-RADS (Breast Imaging Reporting and Data System) [2], are encompassed those conditions such as spiculations, focal retraction and parenchymal edge distortion associated with invasive ductal carcinoma, invasive lobular carcinoma or ductal carcinoma in situ. However, AD could also be associated with benign lesions such as radial scars, complex sclerosing lesions, sclerosing adenosis, fat necrosis, breast fibromatosis and post-procedural change [1]. Hence, it is important to utilize at best the radiological tools in order to exclude malignancy.

Although digital mammography currently represents the standard breast imaging technique for the detection of breast neoplasms, it has the big limit of incurring false positives and/or negatives due to tissue overlapping [3]. Moreover, digital breast tomosynthesis (DBT) is approved by the US Food and Drug Administration to be used in combination with full-field digital mammography (FFDM) [4–8]. Interestingly, architectural distortion is detected more frequently at DBT than at 2D digital mammography and may even be occult at conventional 2D imaging, especially in areas of increased glandular density [5]. The DBT exam, compared to digital mammography, has increased the detection rate of AD which are however more frequently found to be benign alterations; therefore, these findings are putting doctors more frequently in the process of precarious decisions between implementing either a conservative or a more invasive management. DBT emerged as a 3D technique capable of improving the detection of mammary cancers, especially in breasts with a prevalent glandular pattern. To this regard, digital breast tomosynthesis spot compression view (DBT-SCV) may be an additional projection to exploit for confirmation.

The main question that arose following the use of tomosynthesis is whether the quantity of radiation dose is acceptable. According to the European Protocol for the Quality Control of Physical and Technical Aspects of Mammographic Screening [9], a value of about 1.1 mGy was defined as the acceptable dose limit for each single projection, both for FFDM and for DBT (for a glandular thickness of about 4.5 cm). Therefore, the use of the double projection (DBT + FFDM) would lead to an acceptable average glandular dose (AGD) of about 2.2 mGy [9]. Furthermore, we studied the mammary gland performing only DBT, replacing FFDM with synthetic 2D views, obtained with C-view software, with the same image quality [4].

The purpose of this study was to assess if in the architectural distortion detected by DBT, DBT-SCV could be an additional projection to confirm the alteration.

Another aim of the study was to determine the negative predictive value (NPV) of DBT-SCV and the positive predictive value (PPV) of DBT-SCV and DBT. Additionally, we wanted to evaluate associations between DBT, DBT-SCV, US and MR imaging, in terms of BI-RADS score, and histopathologic features of AD. Lastly, we analyzed the radiation dosimetry of DBT, underlining its advantages in relation to the use of the C-view software capable of processing 2D synthetic images.

The key point of the study was to propose a management work flow of architectural distortions that can be easily applied in clinical practice.

## Material and methods

**Fig. 1:**
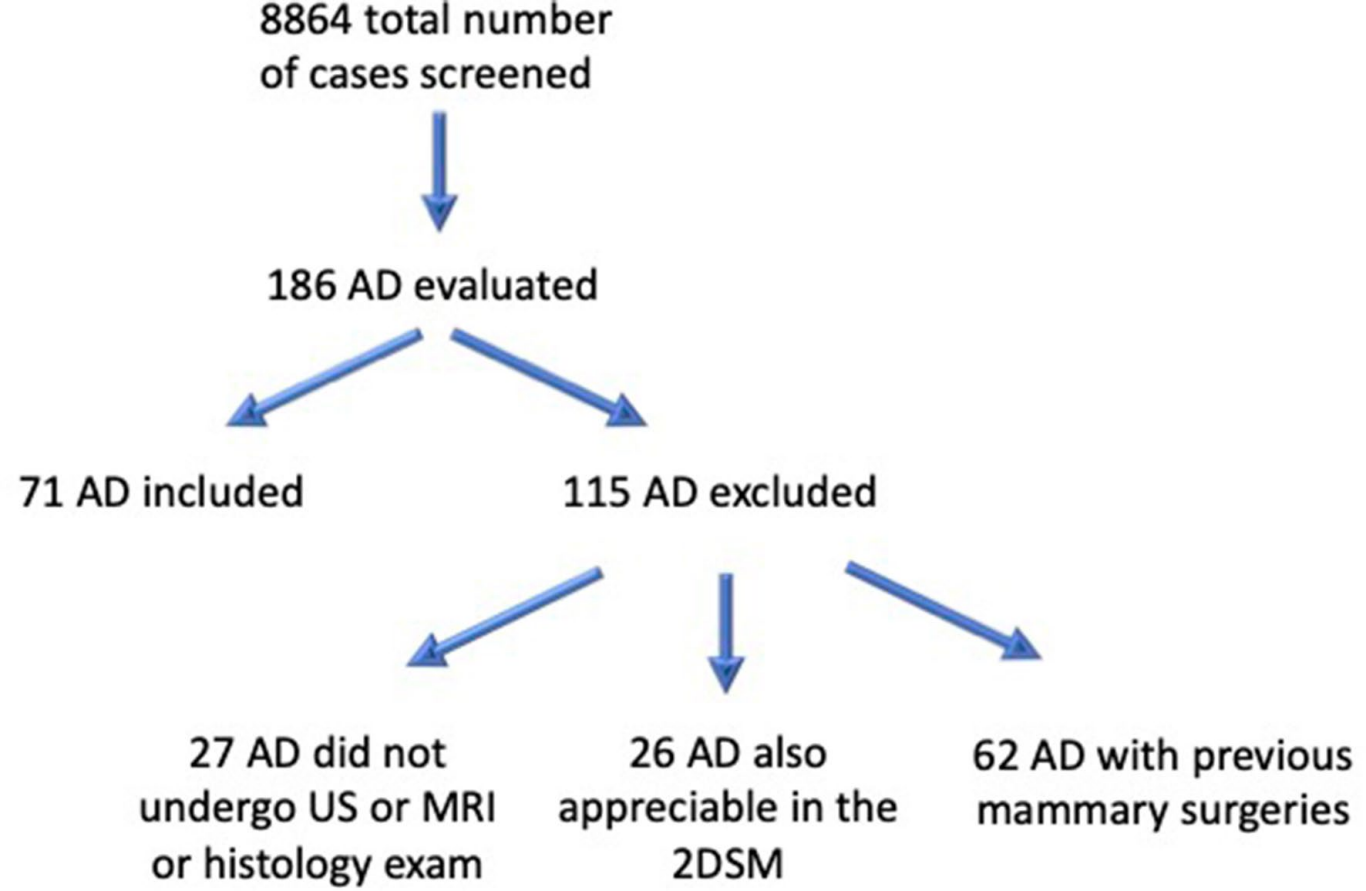
Flow of study enrollment

This was a single-center retrospective database analysis of patients who underwent DBT. Between December 2018 and December 2021, 8864 DBT exams were performed in the University-affiliated clinic European Hospital of Rome. Written informed consent was obtained from all patients, and the study was compliant with the Health Insurance Portability and Accountability Act. We evaluated 186 consecutive cases of AD detected by DBT as primary architectural distortions that arose within the breast de novo, without an underlying identifiable cause. The cases of AD that were also appreciable in two-dimensional synthesized mammography were excluded from the study. Secondary architectural distortions were excluded, and these take into account causes that are known to distort breast tissues, such as breast surgeries including lumpectomy/excision or cosmetic surgeries such as reduction mammoplasty, as well as known traumatic injuries to the breast and invasive breast infections. All AD cases detected by DBT that were not explainable by identifiable cause have undergone additional DBT-SCV to exclude apparent architectural distortions secondary to the overlap phenomenon of normal breast tissue. Only patients who also performed US and MRI examination as well as histological analysis were included in the study (Fig. 1). We have therefore selected 71 ADs found at DBT (0.8%), with patients’ age ranging between 38 and 75 years and an average of 52 years. Figure 1 shows the flow of study enrollment.

The tomosynthesis mammographies were performed both in craniocaudal (CC) and mid-lateral-oblique (MLO) projections with Hologic Selenia TM Dimensions System, using C-View software for interpretation [10–12]. The DBT system determines target material, filtration and tube voltage by compressed breast thickness and adjusts to exposure output (mAs) using an automatic exposure control system to obtain appropriate image density in clinical practice. An automatic exposure control is proposed for entrance surface dose (ESD) and AGD measurements during DBT exposure.

DBT image acquisition was performed in a step‐and‐shoot mode, with less than 10 s acquisition time for each breast. Image reconstruction was performed immediately after image acquisition with a slice thickness of 0.5 mm and a reconstruction time less than 15 s. Patient‐related data, such as age, projection orientation (CC or MLO), compressed breast thickness, compression force, exposure factors, target/filter combination, ESD and AGD, were retrieved directly from the images. The use of tomosynthesis projections only with the synthetic 2D images reconstructed through the C-view software together with the use of DBT and DBT-SCV has led our exams to have average glandular radiation dose values within the allowed limits, according to the protocol for quality control of the physical and technical aspects of mammography screening.

Two expert breast radiologists with ten years of experience each, blinded and individually, reviewed DBT images using the BI-RADS system before and after the DBT-SCV.

BI-RADS was assigned as follows:

- BI-RADS 4–5 for AD with evident tissue attraction, focal retraction, deformation, radiating thin straight lines or spiculations, straightening or thickening of Cooper ligaments and crookedness of the glandular architecture or associated with another finding including asymmetry, microcalcifications and altered mammographic density.
- BI-RADS 3 in case of AD with blurred morphology, blurring of normal tissue planes such as the fat-fibroglandular junction and compression of tissue around, in the absence of evident tissue attraction or deformation or any other associated anomalies.
- BI-RADS 2 in case of morphological distortion and geometric changes or distortion disappearance.

According to the BI-RADS system, each identified specimen is assigned in categories expressing the probability of malignancy [13]. Instrumental guided biopsy could be used to achieve tissue diagnosis: US-guided when there was US correlate, conventional stereotactic biopsy if possible or DBT-guided biopsy if lesions were only visible in the latter. Corresponding BI-RADS score at US / MRI exams and pathology results were reviewed and compared. The PPV of DBT and of DBT-SCV and the NPV of DBT-SCV were evaluated in depicting malignancy of AD.

The average AGD and ESD for each side and projection were calculated, and for analytical simplicity, the patients were divided into six groups (2–3, 3.1–4, 4.1–5, 5.1–6, 6.1–7 and 7.1–8 cm) according to the compressed breast thickness. The reported ESD and AGD values were verified during regular quality assurance measurements, using conversion coefficients reported in the literature [14].

All data were analyzed with a spreadsheet software program (Excel, 2013 version, Microsoft). Data analysis was performed using statistics software SAS Enterprise, version 4.2, SAS Institute. Statistical significance was determined with the Chi-square test for categorical variables or the unpaired t test for continuous variables; *p* values less than 0.05 were considered statistically significant. Cohen’s kappa statistics was calculated for breast radiologists’ correlation degree and for inter-relationships between DBT, DBT-SCV, US and MRI.

## Results

Baseline characteristics, radiological parameters and histopathological results of the included patients are listed in Table 1. The study population comprised of 71 patients with a mean age of 56.5 $\pm$ 18. BI-RADS 3–5 was the most common radiological score in all radiological methods, and radial scars was the most common histological result obtained.

**Table 1:** Baseline characteristics, radiological parameters and histopathological results of the included patients

Total number of patients	71
*Age (in years)*	
Mean	56.5 ± 18
Median	52
Range	38–75
*Histopatological result*	
Invasive ductal carcinoma	13 (18.3%)
Ductal carcinoma in situ	3 (4.2%)
Invasive lobular carcinoma	11 (15.5%)
Radial scars	16 ( 22.5%)
Sclerosing adenosis	12 (17%)
Stromal fibrosis	11 (15.5%)
Adenosis/mastopathy	5 (7%)
*Spot-DBT*	
BI-RADS 3–5	57 (80.3%)
BI-RADS 2	14 (19.7%)
*US*	
BI-RADS 3–5	40 (56.3%)
BI-RADS 2	31 (43.7%)
*MRI*	
BI-RADS 3–5	31 (43.7%)
BI-RADS 2	40 (56.3%)

**Fig. 2:**
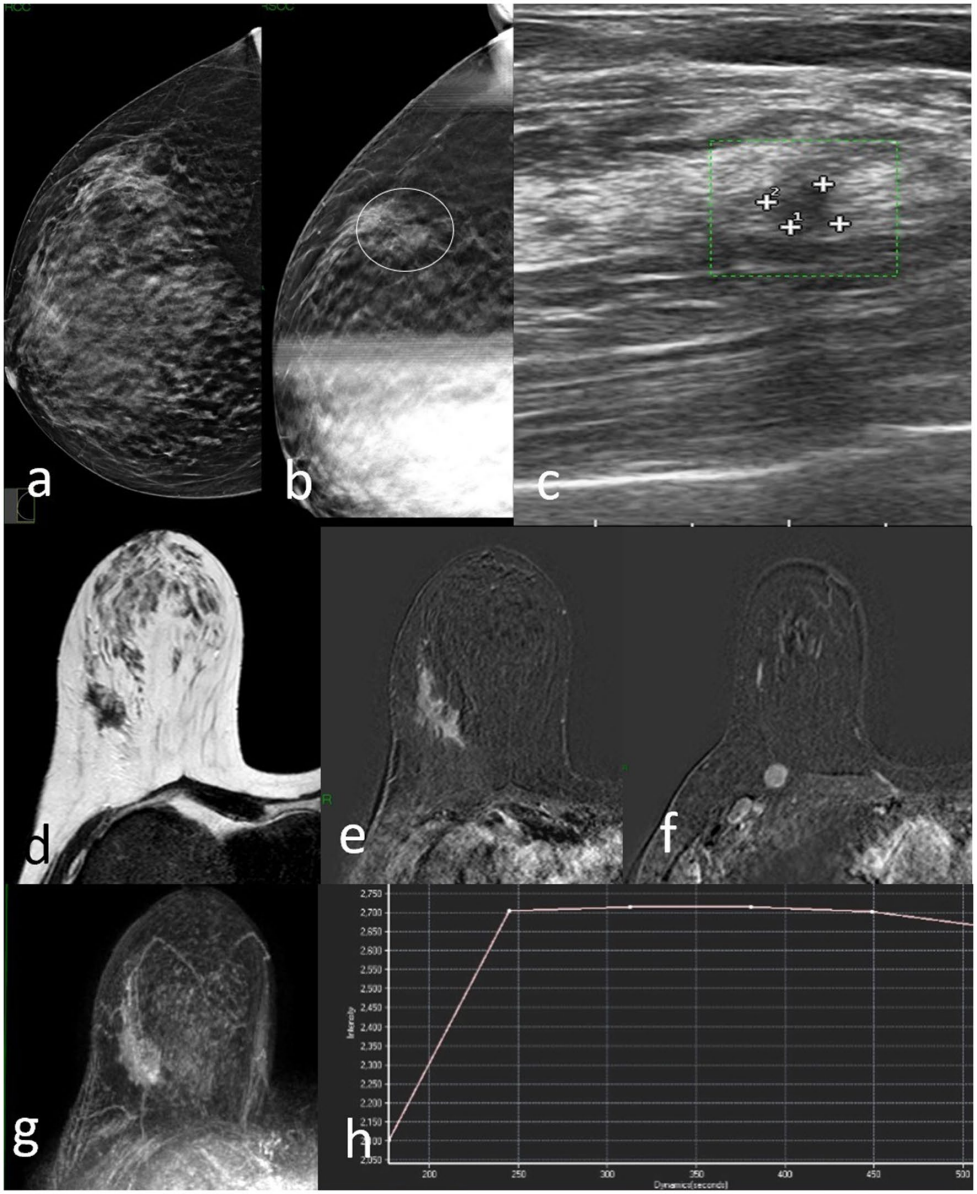
DBT right craniocaudal (**a**) and DBT spot compression (**b**) tomosynthesis mammogram shows an area of architectural distortion with pleomorphic microcalcifications involving the upper outer quadrant (circle). US image shows suspected hypoechoic area (**c**). MRI T2W TSE image detects an irregularly shaped mass with spiculations in upper right breast corresponding to the mammographic image (**d**). Post-contrast subtracted axial fat-suppressed T1-weighted image (**e**) shows heterogeneous internal enhancement pattern and there is also an ipsilateral prepectoral lymphadenopathy (**f**). The right breast finding at maximum intensity projection (MIP) image (**g**); Dynamic MR image reveals a type II T/SI kinetic curve with a rapid initial rise followed by a plateau in the delayed phase (**h**). Biopsy resulted invasive ductal carcinoma at histological examination

**Fig. 3:**
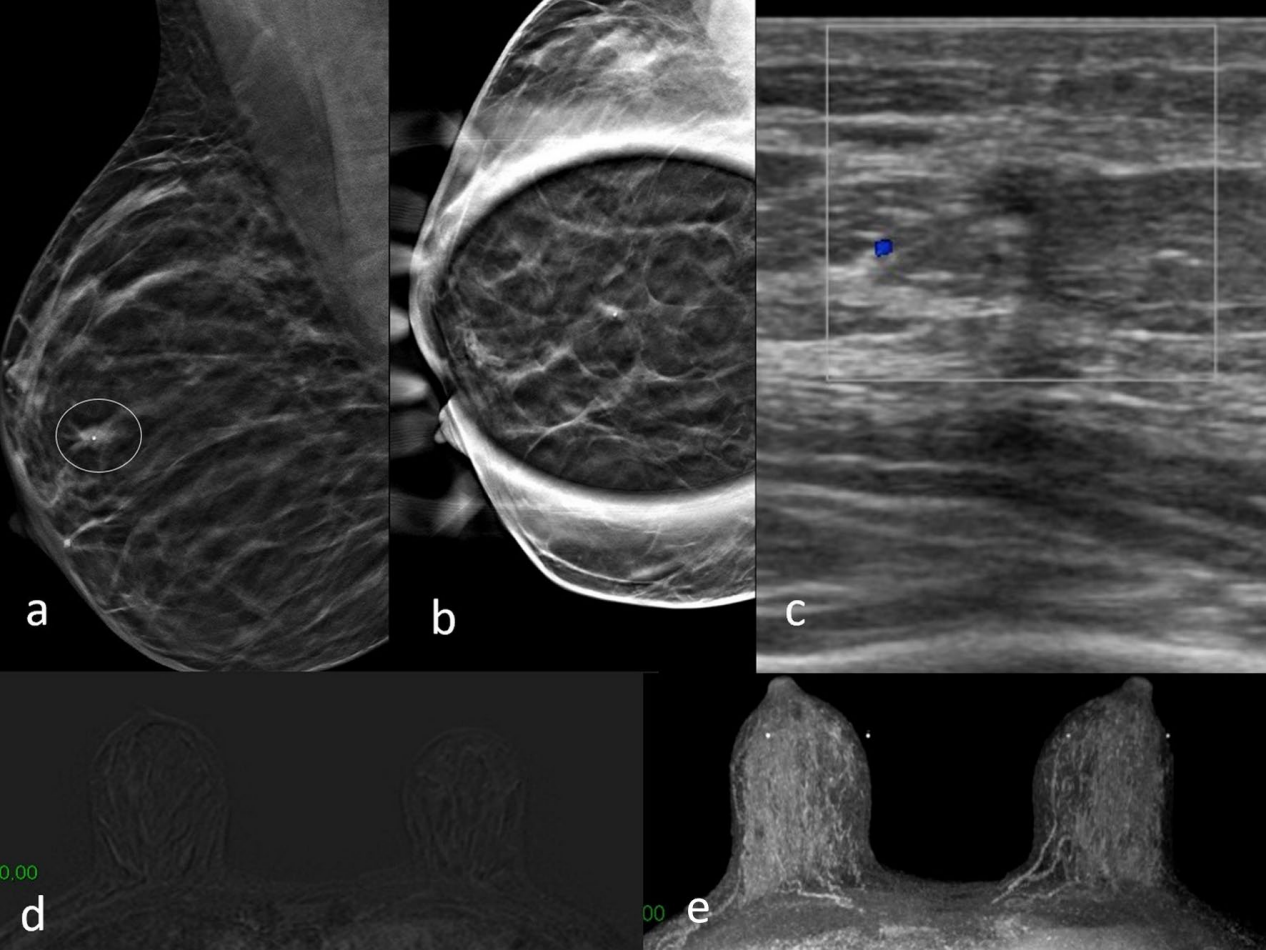
DBT right mediolateral oblique projection **a** shows an area of architectural distortion with a millimeter lump, in the upper periareolar area of right breast (circle). Digital tomosynthesis spot compression view **b** shows only the architectural distortion, the nodular formation is no longer visible. Focused US image of the right upper periareolar area shows irregular hypoechogenic mass corresponding to mammographic finding (**c**). No abnormal enhancement observed in dynamic contrast-enhanced MRI study in subtraction T1-weighted (**d**) and MIP (**e**). Biopsy resulted ductal carcinoma in situ at histological examination

**Fig. 4:**
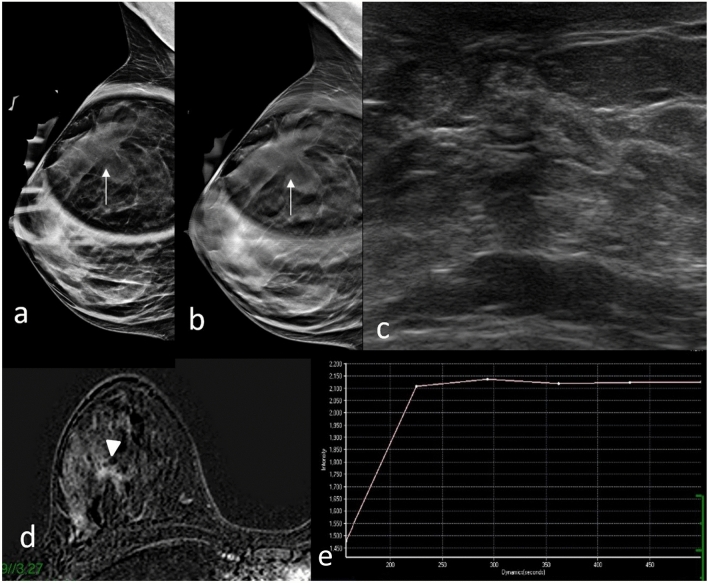
DBT mediolateral oblique spot compression view **a**, **b** shows an area of architectural distortion in upper right breast (arrows). US image shows an area of inhomogeneous echotexture (**c**). Dynamic contrast-enhanced MRI study shows irregular heterogeneous enhancements (arrowhead) in right upper outer quadrant (**d**); signal time-intensity curve obtained by breast dynamic contrast enhancement–MRI: type II time-intensity curve where the signal intensity did not change over time after its initial increase during the delayed phase (**e**). Biopsy resulted radial scar at histological examination

**Fig. 5:**
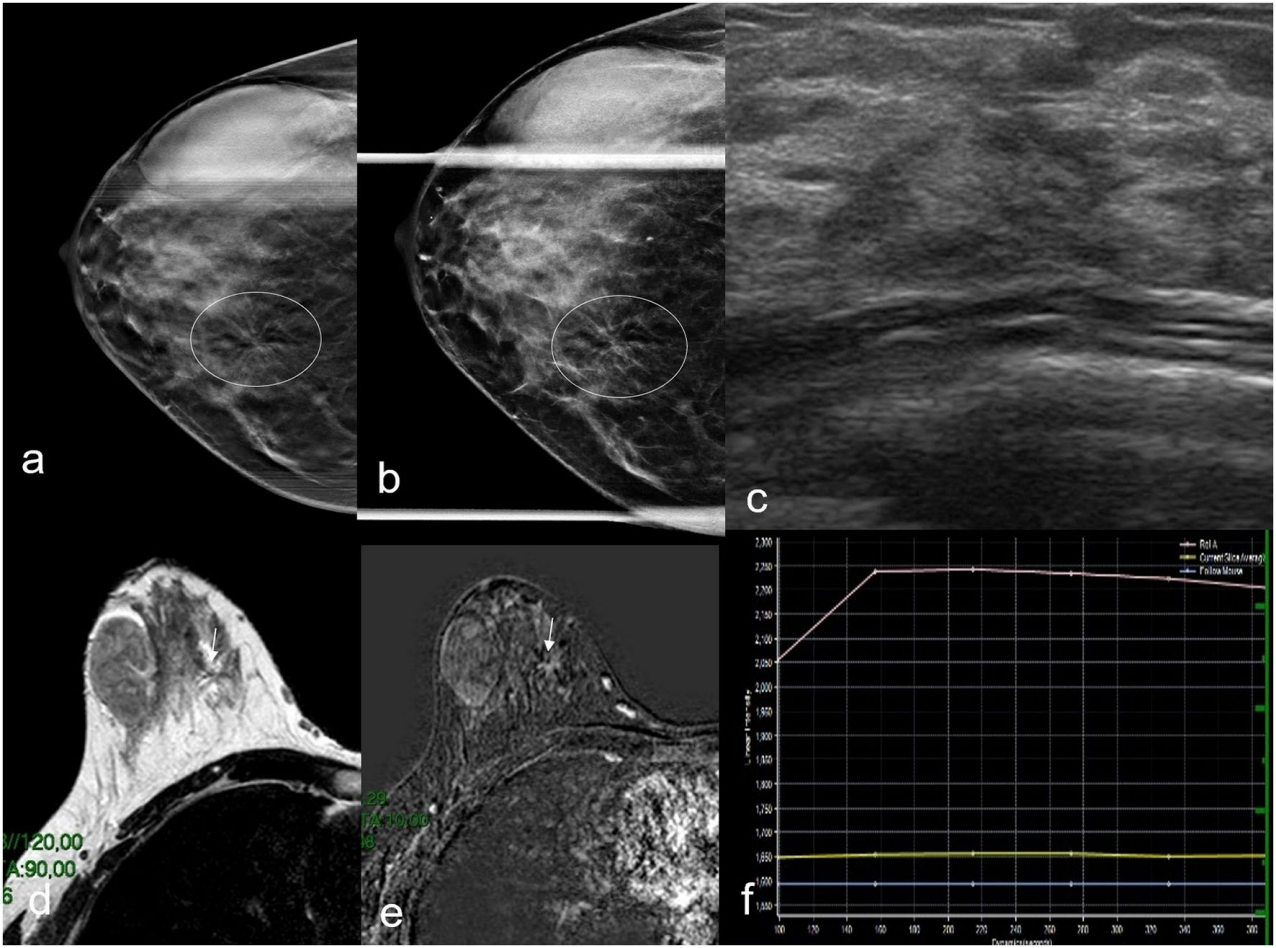
DBT **a** and DBT **b** spot compression in right CC view shows an area of architectural distortion in the inner right breast (circle). No correlation in US examination **c**. Axial T2W **d** and dynamic contrast-enhanced **e** MRI study demonstrates an irregularly shaped mass and enhancement (arrows). Time-intensity curve obtained by breast dynamic contrast enhancement–MRI showed type II time-intensity curve **f**. Biopsy resulted invasive lobular carcinoma at histological examination. Gross fibroadenoma in the outer quadrants known in the medical history is confirmed on every imaging exam

**Fig. 6:**
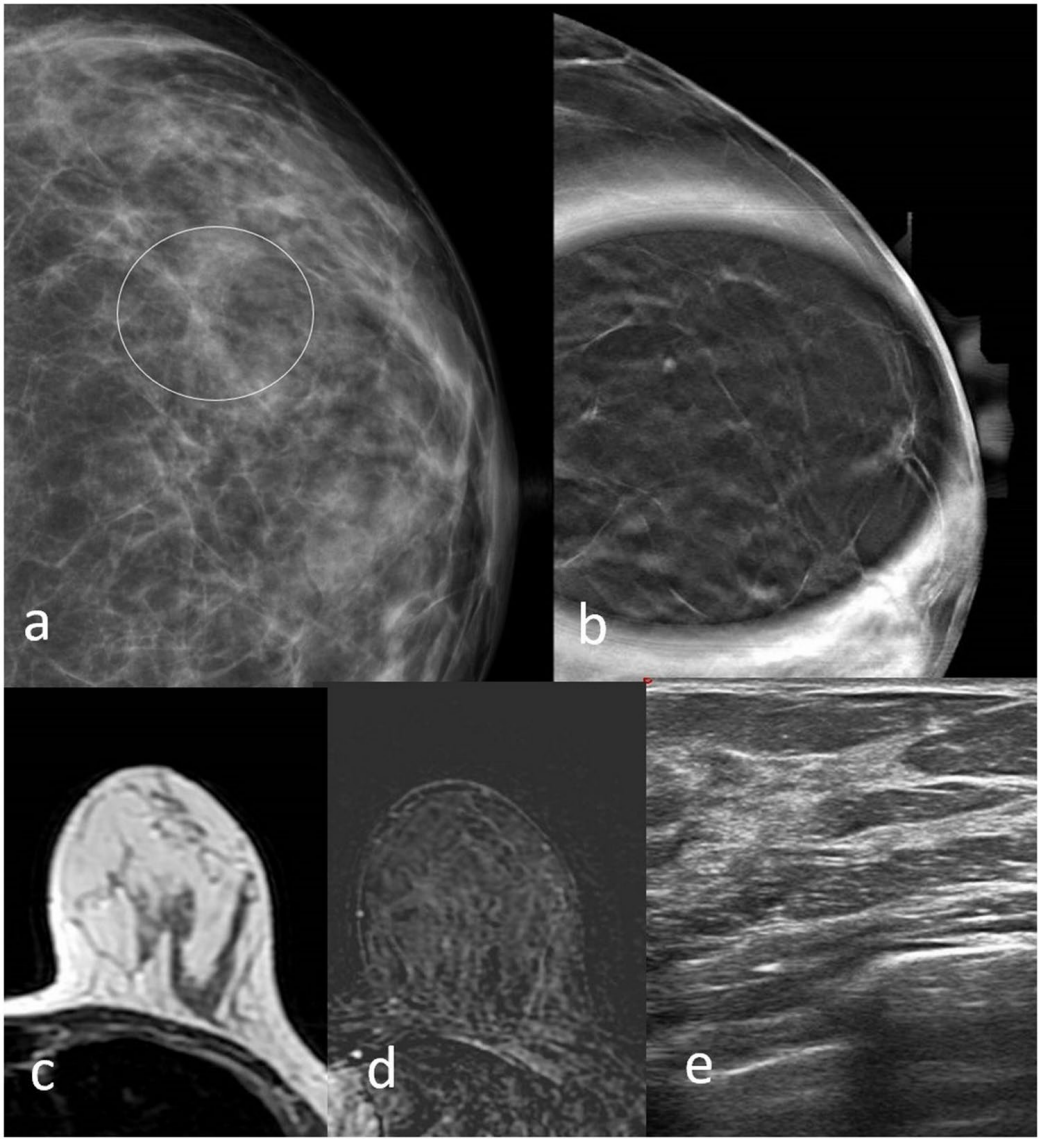
DBT left craniocaudal (RCC) mammography shows an area of architectural distortion (circle) (**a**), which is no longer evident in DBT spot compression mammography (**b**). No correlation is evident on ultrasound (**e**) or MRI examination (**c**, **d**). Biopsy resulted stromal fibrosis at histological examination

Of the 71 AD cases found at DBT, core biopsy yielded malignant pathology in 27 (38%) tissue samples: 13 ductal invasive carcinomas (Fig. 2), three ductal carcinomas in situ (Fig. 3) and 11 invasive lobular carcinomas. Among the 44 non-malignant cases of AD, 28 were considered as high-risk lesions: 16 radial scars (Fig. 4) and 12 complex sclerosing lesion (Fig. 5). Among the 16 non-high-risk causes of benign AD, stromal fibrosis (Fig. 6) was the most common, present in 11 biopsy specimens.

DBT-SCV confirmed and better identified malignancy-related distortion in all cases of cancer except in two cases of carcinoma in situ. Additionally, DBT-SCV showed changes/disappearance of the distortion caused by 38 benign lesions.

The reassessment of BI-RADS regarding the DBT and DBT-SCV images showed very strong agreement between the two breast radiologists, with a Cohen kappa of 0.94 in the benign cases and of 0.98 in the malignant cases. Of the 27 ADs that were ultimately characterized as malignant, 25 were assigned DBT-SCV BI-RADS score of 4 or 5 (vs 21 considering DBT BI-RADS), and in two cases of carcinoma, in situ DBT-SCV BI-RADS score was 2 and 3 (vs DBT BI-RADS score 3 and 4, respectively). Thirty-eight ADs that resulted to be benign lesions at histology were assigned DBT-SCV BI-RADS 2 or 3, compared to DBT BI-RADS 3 or 4. However, in five benign ADs, DBT-SCV highlighted a BI-RADS score of 4 in agreement with DBT, and score of 5 in one AD that was considered BI-RADS 4 on DBT (Table 2).

**Table 2:** Correlation between DBT BI-RADS, DBT-SCV BI-RADS, US BI-RADS and MRI BI-RADS score in benign architectural distortions

Patient	Histology	DBT	DBT-SCV	US	MRI
1	Radial scars	BI-RADS 4	BI-RADS 5	BI-RADS 4	BI-RADS 4
2	Radial scars	BI-RADS 4	BI-RADS 4	BI-RADS 4	BI-RADS 3
3	Radial scars	BI-RADS 4	BI-RADS 4	BI-RADS 4	BI-RADS 2
4	Radial scars	BI-RADS 3	BI-RADS 2	BI-RADS 2	BI-RADS 2
5	Radial scars	BI-RADS 4	BI-RADS 3	BI-RADS 3	BI-RADS 3
6	Radial scars	BI-RADS 4	BI-RADS 3	BI-RADS 2	BI-RADS 2
7	Radial scars	BI-RADS 4	BI-RADS 2	BI-RADS 3	BI-RADS 2
8	Radial scars	BI-RADS 4	BI-RADS 3	BI-RADS 4	BI-RADS 3
9	Radial scars	BI-RADS 4	BI-RADS 4	BI-RADS 4	BI-RADS 3
10	Radial scars	BI-RADS 4	BI-RADS 2	BI-RADS 3	BI-RADS 2
11	Radial scars	BI-RADS 3	BI-RADS 3	BI-RADS 3	BI-RADS 2
12	Radial scars	BI-RADS 4	BI-RADS 3	BI-RADS 4	BI-RADS 2
13	Radial scars	BI-RADS 3	BI-RADS 2	BI-RADS 3	BI-RADS 2
14	Radial scars	BI-RADS 4	BI-RADS 4	BI-RADS 4	BI-RADS 2
15	Radial scars	BI-RADS 4	BI-RADS 3	BI-RADS 2	BI-RADS 2
16	Radial scars	BI-RADS 3	BI-RADS 2	BI-RADS 3	BI-RADS 2
17	Sclerosing adenosis	BI-RADS 4	BI-RADS 4	BI-RADS 2	BI-RADS 2
18	Sclerosing adenosis	BI-RADS 3	BI-RADS 2	BI-RADS 2	BI-RADS 3
19	Sclerosing adenosis	BI-RADS 4	BI-RADS 3	BI-RADS 4	BI-RADS 2
20	Sclerosing adenosis	BI-RADS 4	BI-RADS 3	BI-RADS 2	BI-RADS 2
21	Sclerosing adenosis	BI-RADS 4	BI-RADS 3	BI-RADS 2	BI-RADS 2
22	Sclerosing adenosis	BI-RADS 4	BI-RADS 3	BI-RADS 2	BI-RADS 2
23	Sclerosing adenosis	BI-RADS 4	BI-RADS 3	BI-RADS 2	BI-RADS 2
24	Sclerosing adenosis	BI-RADS 4	BI-RADS 3	BI-RADS 2	BI-RADS 2
25	Sclerosing adenosis	BI-RADS 3	BI-RADS 3	BI-RADS 2	BI-RADS 2
26	Sclerosing adenosis	BI-RADS 4	BI-RADS 3	BI-RADS 2	BI-RADS 2
27	Sclerosing adenosis	BI-RADS 3	BI-RADS 2	BI-RADS 2	BI-RADS 2
28	Sclerosing adenosis	BI-RADS 4	BI-RADS 3	BI-RADS 2	BI-RADS 2
29	Stromal fibrosis	BI-RADS 4	BI-RADS 3	BI-RADS 2	BI-RADS 2
30	Stromal fibrosis	BI-RADS 4	BI-RADS 2	BI-RADS 2	BI-RADS 2
31	Stromal fibrosis	BI-RADS 4	BI-RADS 3	BI-RADS 2	BI-RADS 2
32	Stromal fibrosis	BI-RADS 4	BI-RADS 3	BI-RADS 2	BI-RADS 2
33	Stromal fibrosis	BI-RADS 4	BI-RADS 3	BI-RADS 2	BI-RADS 2
34	Stromal fibrosis	BI-RADS 4	BI-RADS 3	BI-RADS 2	BI-RADS 2
35	Stromal fibrosis	BI-RADS 4	BI-RADS 3	BI-RADS 2	BI-RADS 2
36	Stromal fibrosis	BI-RADS 4	BI-RADS 3	BI-RADS 2	BI-RADS 2
37	Stromal fibrosis	BI-RADS 4	BI-RADS 3	BI-RADS 2	BI-RADS 2
38	Stromal fibrosis	BI-RADS 4	BI-RADS 3	BI-RADS 2	BI-RADS 2
39	Stromal fibrosis	BI-RADS 4	BI-RADS 3	BI-RADS 2	BI-RADS 2
40	Adenosis / Mastopathy	BI-RADS 3	BI-RADS 2	BI-RADS 2	BI-RADS 2
41	Adenosis / Mastopathy	BI-RADS 3	BI-RADS 2	BI-RADS 2	BI-RADS 2
42	Adenosis / Mastopathy	BI-RADS 3	BI-RADS 2	BI-RADS 2	BI-RADS 2
43	Adenosis / Mastopathy	BI-RADS 3	BI-RADS 2	BI-RADS 2	BI-RADS 2
44	Adenosis / Mastopathy	BI-RADS 3	BI-RADS 2	BI-RADS 2	BI-RADS 2

The PPV of architectural distortion for malignancy at DBT was 38.5%, while on DBT-SCV was 64%, highlighting a statistically significant result (*p* < 0.05). The NPV of architectural distortion for malignancy on DBT-SCV was 94%.

Importantly, the difference between DBT and DBT-SCV in the detection of benign ADs was statistically significant (*p* < 0.05), while it was not significant in the detection of malignant ADs (*p* > 0.05).

In cases of malignancy, the BI-RADS score calculated at DBT-SCV differed from that of MRI in eight cases (3/13 invasive ductal carcinoma, 2/3 carcinoma in situ, 3/11 invasive lobular carcinoma), while BI-RADS at DBT-SCV differed from US in only four cases (0 invasive ductal carcinoma, 2 carcinoma in situ, 2 invasive lobular carcinoma) (Table 3).

**Table 3:** Correlation between DBT BI-RADS, DBT-SCV BI-RADS, US BI-RADS and MRI BI-RADS score in malignant architectural distortions

Patient	Histology	DBT	DBT-SCV	US	MRI
1	Invasive ductal carcinoma	BI-RADS 5	BI-RADS 5	BI-RADS 5	BI-RADS 5
2	Invasive ductal carcinoma	BI-RADS 4	BI-RADS 4	BI-RADS 4	BI-RADS 5
3	Invasive ductal carcinoma	BI-RADS 4	BI-RADS 4	BI-RADS 4	BI-RADS 5
4	Invasive ductal carcinoma	BI-RADS 3	BI-RADS 4	BI-RADS 4	BI-RADS 5
5	Invasive ductal carcinoma	BI-RADS 4	BI-RADS 4	BI-RADS 4	BI-RADS 4
6	Invasive ductal carcinoma	BI-RADS 4	BI-RADS 4	BI-RADS 4	BI-RADS 4
7	Invasive ductal carcinoma	BI-RADS 4	BI-RADS 4	BI-RADS 4	BI-RADS 4
8	Invasive ductal carcinoma	BI-RADS 4	BI-RADS 4	BI-RADS 4	BI-RADS 4
9	Invasive ductal carcinoma	BI-RADS 3	BI-RADS 4	BI-RADS 4	BI-RADS 4
10	Invasive ductal carcinoma	BI-RADS 4	BI-RADS 4	BI-RADS 4	BI-RADS 4
11	Invasive ductal carcinoma	BI-RADS 4	BI-RADS 4	BI-RADS 4	BI-RADS 4
12	Invasive ductal carcinoma	BI-RADS 4	BI-RADS 4	BI-RADS 4	BI-RADS 4
13	Invasive ductal carcinoma	BI-RADS 4	BI-RADS 4	BI-RADS 4	BI-RADS 4
14	Ductal carcinoma in situ	BI-RADS 3	BI-RADS 2	BI-RADS 4	BI-RADS 2
15	Ductal carcinoma in situ	BI-RADS 4	BI-RADS 3	BI-RADS 4	BI-RADS 4
16	Ductal carcinoma in situ	BI-RADS 4	BI-RADS 4	BI-RADS 4	BI-RADS 2
17	Invasive lobular carcinoma	BI-RADS 4	BI-RADS 5	BI-RADS 2	BI-RADS 4
18	Invasive lobular carcinoma	BI-RADS 3	BI-RADS 4	BI-RADS 3	BI-RADS 4
19	Invasive lobular carcinoma	BI-RADS 4	BI-RADS 4	BI-RADS 4	BI-RADS 4
20	Invasive lobular carcinoma	BI-RADS 4	BI-RADS 4	BI-RADS 4	BI-RADS 4
21	Invasive lobular carcinoma	BI-RADS 4	BI-RADS 4	BI-RADS 4	BI-RADS 4
22	Invasive lobular carcinoma	BI-RADS 4	BI-RADS 4	BI-RADS 4	BI-RADS 4
23	Invasive lobular carcinoma	BI-RADS 3	BI-RADS 4	BI-RADS 4	BI-RADS 5
24	Invasive lobular carcinoma	BI-RADS 4	BI-RADS 5	BI-RADS 5	BI-RADS 4
25	Invasive lobular carcinoma	BI-RADS 4	BI-RADS 4	BI-RADS 4	BI-RADS 4
26	Invasive lobular carcinoma	BI-RADS 3	BI-RADS 4	BI-RADS 4	BI-RADS 4
27	Invasive lobular carcinoma	BI-RADS 4	BI-RADS 4	BI-RADS 4	BI-RADS 4

On the other hand, in case of benignity, only 14 BI-RADS cases detected at DBT-SCV correlated with MRI (7/16 radial scars, 1/12 sclerosing adenosis, 1/11 stromal fibrosis, 5/5 adenosis mastopathy), while 15 BI-RADS cases detected at DBT-SCV correlated with US (7 radial scars, 2 sclerosing adenosis, 1 stromal fibrosis, 5 adenosis mastopathy) (Table 2). Architectural distortion without a US/MRI correlate was less likely to represent malignancy than architectural distortion with a correlate (*p* < 0.05), in fact, benign AD was significantly associated with MRI-negative correlation (*p* < 0.05).

Moreover, in assessing ADs with malignant histology, DBT, DBT-SCV, US and MRI are strongly correlated with the analysis of Cohen’s kappa statistics for inter-relationships (0.89, 0.96 and 0.91, respectively); additionally, a very strong correlation is appreciable between DBT-SCV, US and MRI (0.92 and 0.96, respectively) and between US and MRI (0.91) (Table 4).

**Table 4:** Cohen’s kappa statistics inter-relationships of DBT, DBT-SCV, US and MRI in the malignant cases

	DBT	DBT-SCV	US	MRI
DBT	1			
DBT-SCV	0.89	1		
US	0.96	0.92	1	
MRI	0.91	0.96	0.91	1

On the other hand, in ADs with benign histology, in the analysis of Cohen’s kappa statistics for inter-relationships, DBT is weakly correlated with DBT-SCV (0.40), and negatively correlated with US and MRI (< 0); a poor correlation is appreciable between DBT-SCV, US and MRI (0.25, 0.66, respectively); lastly, a very strong correlation is highlighted between US and MRI (0.85) (Table 5).

**Table 5:** Cohen’s kappa statistics inter-relationships of DBT, DBT-SCV, US and MRI in the benign cases

	DBT	DBT-SCV	US	MRI
DBT	1			
DBT-SCV	0.40	1		
US	< 0	0.25	1	
MRI	< 0	0.66	0.85	1

The radiation dose evaluation carried out in our study revealed the following average dose data for a single DBT projection at different compression thicknesses and were compared with the standard data defined by the reference protocols. (For a glandular thickness of about 2–3 cm, we reported AGD mean of 0.54–0.71 mGy and 0.68–0.57 mGy for each DBT view with 0.73 mGy for DBT-SCV in relation to a defined acceptable dose less than 1.5 mGy; for a glandular thickness of about 4.1–5 cm, we reported AGD mean of 1.19–1.21 mGy and 1.23–1.18 mGy for each DBT view with 1.22 mGy for DBT-SCV in relation to a defined acceptable dose less than 2.5 mGy; for a glandular thickness of about 7.1–8 cm, we reported AGD average of 2.29–2.68 mGy and 2.88–2.94 mGy for each DBT view with 2.04 mGy for DBT-SCV in relation to a defined acceptable dose less than 6.5 mGy (Table 6).

**Table 6:** Dose rates for each tomosynthesis mammography projection in comparison with European guidelines acceptable dose reference values

Breast thickness	AGD mean		AGD mean		DBT-SCV	European guidelines	ESD mean		ESD mean		DBT-SCV
	Right		Left			Acceptable dose	Right		Left		
	CC	MLO	CC	MLO			CC	MLO	CC	MLO	
2–3	0.54	0.71	0.68	0.57	0.73	< 1.5	2.64	2.61	2.74	3.28	3.4
3.1–4	0.97	1.01	0.88	0.98	0.94	< 2.0	3.4	4.99	3.28	4.73	3.72
4.1–5	1.19	1.21	1.23	1.18	1.22	< 2.5	3.66	5.03	4.03	4.74	4.54
5.1–6	1.42	1.45	1.39	1.31	1.34	< 3	4.33	4.34	4.34	3.95	4.57
6.1–7	1.65	1.74	1.68	1.72	1.52	< 4.5	5.06	5.26	4.99	4.73	5.02
7.1–8	2.29	2.68	2.88	2.94	2.04	< 6.5	4.66	5.44	5.21	4.98	5.66

## Discussion

Compared to tomosynthesis, architectural distortion is detected less frequently at 2D digital mammography, in which it may even be occult. In fact, at conventional 2D screening mammography, 12–45% of missed breast cancers are retrospectively distinguished as areas of architectural distortion [15]. Tomosynthesis reduces the structured noise that limits 2D mammography, thanks to its ability in eliminating the superimposed tissues [15].

Our data (0.8%) that consider AD to be suspicious or suggestive of malignancy are slightly different compared to what reported by Bahl M. et al. (0.2%) [16]. The small variation could be due to the smaller number of total examinations we performed. Partyka L. al., have shown that the architectural distortion is more easily identified with tomosynthesis than with 2D mammography [5], with 73% of identified distortions seen at tomosynthesis only, of which 21% yielding a cancer diagnosis. Few studies have focused on tomosynthesis-detected architectural distortions to date, and optimal clinical management of these distortions still awaits. Skaane P. et al. in their prospective clinical screening trial comparing 2D digital mammography with DBT demonstrated a 15% reduction in the recall rate and a 27% increase in cancer detection rate, with a 40% increase in the detection of invasive tumors [17]; similar results were obtained by Friedewald et al. [18].

The use of focused compression performed with tomosynthesis can make the distortion more evident [3], owing to the combined advantages of DBT and SCV in clearing away superimposed tissue at imaging [15]; hence, it can be considered as a valid aid in the diagnosis of AD in 3D mammography. Durand et al., argue that spot compression tomosynthesis may also be useful in subtle cases of distortion or when the distortion is visible only on a single view [15]. Our study shows that in 71 cases of AD identified at DBT examination, DBT-SCV highlights findings of malignancy, with BI-RADS score of 4 or 5, in 25 cases out of the 27 with malignant histology diagnosis; interestingly, six confirmed malignant ADs cases were considered BI-RADS 3 on DBT (Table 3). Moreover, digital tomosynthesis spot compression view showed changes/disappearance of the distortion in 38 out of the 44 benign cases. Only in two cases of ductal invasive carcinoma, DBT-SCV demonstrated the resolution of AD. It is important to be aware that subtle architectural distortions may mimic normal fibroglandular tissue on spot compression views due to the fact that some cancers can “spot away” or be less prominent. Therefore, a suspicious architectural distortion seen on standard, full-field CC or MLO tomosynthesis images may require additional workup even if the spot compression views appear unremarkable [15]. Durand M.A. et al. affirm that an AD suspected on DBT, or recognizable in a single tomosynthesis view, can benefit from confirmation by DBT-SCV [15]. The same authors state that some ADs mimicking the fibroglandular component of the breast may disappear at DBT-SCV; this does not change the fact that suspicious ADs necessitate further screening.

Our study shows that of the 27 ADs having malignant histology, 25 received a BI-RADS 4 or 5 at DBT-SCV (vs 21 on DBT), while of the 44 benign ADs, only six received BI-RADS score of 4 or 5 on DBT-SCV (vs 32 cases on DBT). Therefore, the calculated PPV of architectural distortion for malignancy at DBT was 38.5%, in agreement with the PPV of 34.6% reported by Choudhery et al. [19]. Also the PPV of architectural distortion for malignancy at DBT-SCV was 64%, comparable with the PPV of 74% reported by Bahl et al. on digital mammography [16]. The PPV of architectural distortion for malignancy on DBT-SCV compared to the PPV on DBT was statistically significant. A very high NPV of architectural distortion for malignancy on DBT-SCV was 94% in our study. To this regard, no data relating to NPV are currently available in the literature.

Increased PPV and high NPV of DBT-SCV represents an encouraging result to promote the use of the DBT-SCV in clinical practice. In our study, the 71 patients were then subjected to complementary ultrasound to check if there was a morphological correlation to DBT and MRI examination, in order to check for the presence of any pathological contrast enhancement and for biopsy sampling guidance (by tomosynthesis guide, under stereotactic guide or ultrasound-guided) for adequate diagnosis of nature. While on the one hand, DBT-SCV, US and MRI strongly correlated with assessing ADs with malignant histology, on the other hand, regarding ADs with benign histology, no correlation was seen between DBT, US and MRI (< 0), while a moderate correlation was appreciable between DBT-SCV, US and MRI (0.25 and 0.66, respectively) (Table 5). These results strengthen the fact that DBT-SCV gives an additional value compared to DBT alone, as it is useful in wiping out AD thus reducing false positives; it could therefore be considered as a follow-up method alternatively to useless biopsy.

In our study, architectural distortion without a US/MRI correlate was less likely to represent malignancy than AD with a correlate (*p* < 0.05); sonographic [15–21] and MRI [22] correlation also emerged in the literature. All ADs require an ultrasound check; in fact AD at DBT is less likely to represent malignancy without correlative findings on US [21]; nonetheless, it is still a prerequisite to undergoing biopsy, given that the risk of malignancy is nearly 30% [21, 23]. Therefore, data available in the literature shows that AD with an ultrasound correlate has a higher probability of malignancy [24].

All 71 patients were subjected to MRI examination because US represents an excellent problem-solving tool useful to exclude malignancy in AD and to avoid unnecessary interventional procedures [25]. MRI negative correlation was significantly associated with benign outcomes [22, 25]. Architectural distortion without MRI correlate could be due to poor lesion neoangiogenesis [25].

If a tomosynthesis-detected architectural distortion has no US or MRI imaging correlate, the following options may be considered at the radiologist’s discretion: tomosynthesis-guided needle localization, tomosynthesis-guided stereotactic biopsy or short-interval follow-up via tomosynthesis [15]. For example, when there is no ultrasound correlation, it is necessary to proceed to histological characterization via tomosynthesis-guided stereotactic biopsy, if available [26, 27], or by MRI-guided biopsy when there is a corresponding pathological enhancement at MRI exam.

In our study, all 71 patients with suspected AD underwent a conventional stereotactic biopsy or tomosynthesis-guided biopsy if lesions were only DBT visible. While the management of suspected AD with a clear counterpart in US and/or MRI is clear in the literature, there are no flawless guidelines regarding the management of AD without US or MRI imaging correlate [15]. Therefore, while DBT-SCV would not have changed the management of malignant lesions, it could indeed have changed that of the benign AD, introducing the possibility to undergo follow-up and avoiding unnecessary biopsy.

**Fig. 7:**
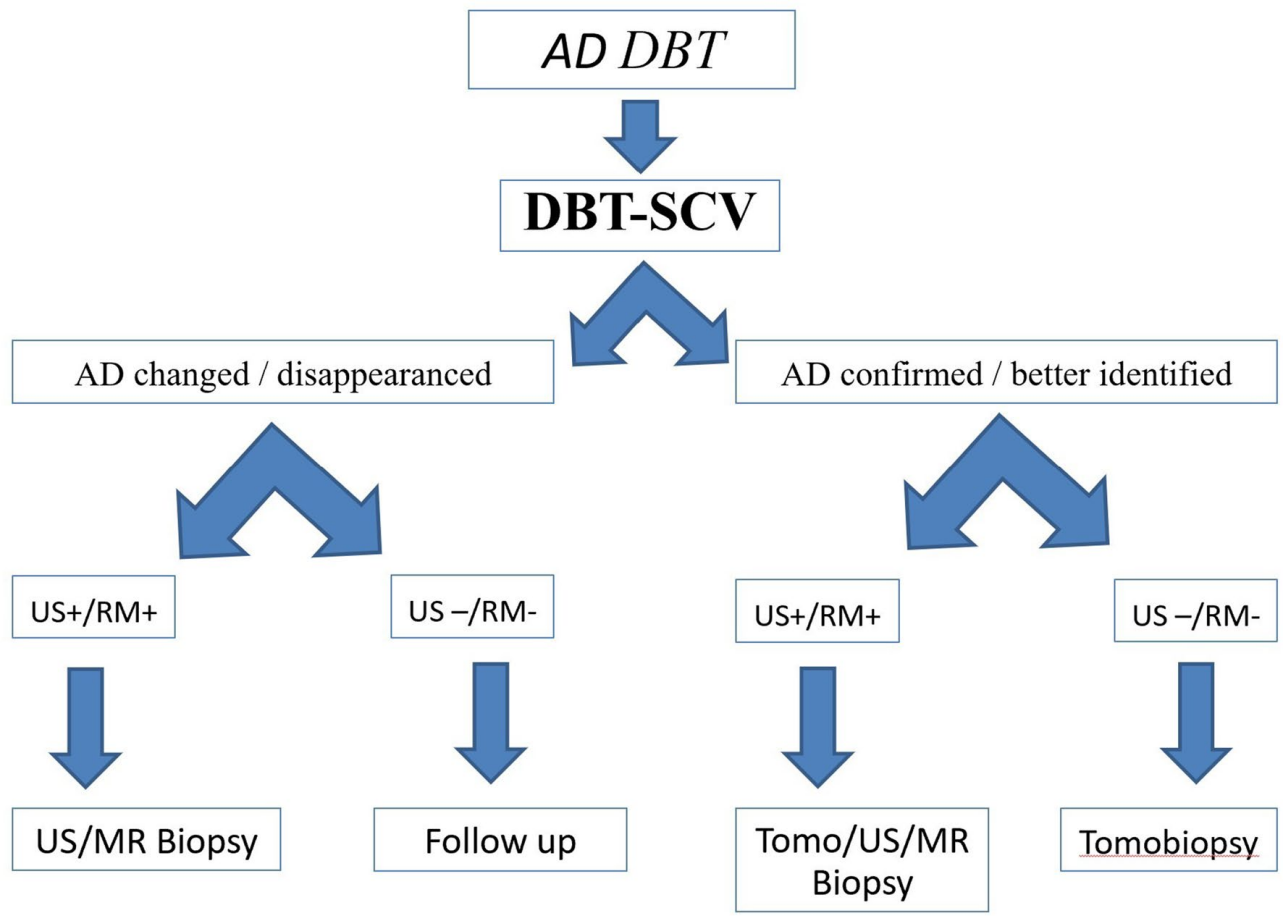
AD–DBT management diagram in clinical practice

Based on the results obtained, we would like to propose a management work-flow in glandular architecture distortions documented at mammography tomosynthesis in clinical practice. Firstly, we would like to stress the fact that appreciable glandular distortions in tomosynthesis should also be re-evaluated with targeted compression in tomosynthesis. In fact, if the AD is confirmed or appears better defined at spot compression view in addition to a positive US/MRI correlation, a biopsy must be taken with an instrumental approach most convenient or accessible to the operator. Furthermore, in case of absence of AD confirmation at US/MRI that had been found however at spot compression, the tomobiopsy should be the preferred approach. On the contrary, if the glandular distortion at the spot compression view changes or disappears, with US/MRI positivity, we can proceed anyway to biopsy; otherwise, in the absence of US/MRI positivity, we can decide toward a follow-up approach (Fig. 7). Thus, in low-risk patients, in the absence of positive anamnestic data for previous oncological disease, AD on DBT without a confirmation in the other imaging methods could be considered probably benign (BI-RADS 3), associated with possible expression of glandular artifacts, hence potentially manageable only with follow-up at radiologist’s discretion.

All patients in our study were subjected to an exclusive study with DBT, using the C-view software for the reconstruction of synthetic 2D images, thus obviating the joint use of DBT with FFDM. Replacing FFDM with synthetic 2D views (reconstructed from the DBT acquisitions) reduced the dose approximately by half to a level that was roughly comparable to that of FFDM [28, 29]. All 71 patients had dose values for CC, MLO and DBT projections absolutely in accordance with the literature [9]; additionally, the dose summation of CC or MLO projections and DBT-SCV was kept within the same range of acceptable doses (Table 6).

In our study, we analyzed AGD as an expression of the absorbed breast dose considering the glandular component as the most radiosensitive tissue of the breast and ESD in correlation with the different breast thicknesses. The use of DBT-SCV remained within the dose limits set by the European Protocol for the Screening and Diagnosis of Breast Cancer proving to be advantageous [9]. Our study presents satisfactory results in characterizing breast lesions at lower radiation doses in relation to various factors such as the type of mammography used, thickness, breast density and a decreased number of patients undergoing examination. Indeed, the AGD received by patients during a single-view DBT acquisition was slightly lower compared to a two-view FFDM acquisition for different breast thicknesses [9]. According to the previous studies [30], 31, there was a statistically significant difference between AGD deriving from mammography machine and calculated AGD. The previous studies published AGD dosimetric data for DBT of 1.74 mGy (*n* = 179) to 2.56 mGy (*n* = 300) [28, 30, 32]. The reported average AGD values both depend on the vendor-specific technical implementation to achieve an optimum between image quality and radiation dose, as well as the breast thickness distribution of the population under study [33].

Study limitations include the narrow population of patients and the retrospective origin of the study. Secondly, results were obtained in a single center and using a single mammographer, Hologic Selenia TM Dimensions System. Future studies are encouraged to extend the population sample analyzed in addition to implementing different mammography manufacturers.

## Conclusion

This study showed that DBT-SCV has a high NPV and a statistically significant higher PPV compared to DBT alone, hence could be useful in the daily practice workflow as an additional projection to better characterize the architectural distortions identified with DBT and improve the management accordingly within the radiation dose limits.

In summary, if a tomosynthesis-detected architectural distortion, without US or MRI correlate, that is neither confirmed nor changed by DBT-SCV, other options such as “wait and see” approach can be taken into consideration in order to reduce unnecessary biopsy.

## References

[1] Gaur S (2013). Architectural distortion of the breast. AJR Am J Roentgenol.

[2] Meucci R (2020). MR imaging-guided vacuum assisted breast biopsy: radiological-pathological correlation and underestimation rate in pre-surgical assessment. Eur J Radiol Open.

[3] Choi Y (2019). Quantitative analysis of radiation dosage and image quality between digital breast tomosynthesis (DBT) with two-dimensional synthetic mammography and full-field digital mammography (FFDM). Clin Imaging.

[4] Zuley ML (2014). Comparison of two-dimensional synthesized mammograms versus original digital mammograms alone and in combination with tomosynthesis images. Radiology.

[5] Partyka L, Lourenco AP, Mainiero MB (2014). Detection of mammographically occult architectural distortion on digital breast tomosynthesis screening: initial clinical experience. AJR Am J Roentgenol.

[6] Peppard HR (2015). Digital breast tomosynthesis in the diagnostic setting: indications and clinical applications. Radiographics.

[7] Korhonen KE (2016). Strategies to increase cancer detection: review of true-positive and false-negative results at digital breast tomosynthesis screening. Radiographics.

[8] Roth RG (2014). Digital breast tomosynthesis: lessons learned from early clinical implementation. Radiographics.

[9] Perry N (2008). European guidelines for quality assurance in breast cancer screening and diagnosis. Fourth edition–summary document. Ann Oncol.

[10] Yun SJ (2017). Benefit of adding digital breast tomosynthesis to digital mammography for breast cancer screening focused on cancer characteristics: a meta-analysis. Breast Cancer Res Treat.

[11] Caumo F (2018). Digital breast tomosynthesis with synthesized two-dimensional images versus full-field digital mammography for population screening: outcomes from the Verona screening program. Radiology.

[12] Bernardi D (2016). Breast cancer screening with tomosynthesis (3D mammography) with acquired or synthetic 2D mammography compared with 2D mammography alone (STORM-2): a population-based prospective study. Lancet Oncol.

[13] Spak DA (2017). BI-RADS. Diagn Interv Imaging.

[14] Gennaro G, Bernardi D, Houssami N (2018). Radiation dose with digital breast tomosynthesis compared to digital mammography: per-view analysis. Eur Radiol.

[15] Durand MA (2016). Tomosynthesis-detected architectural distortion: management algorithm with radiologic-pathologic correlation. Radiographics.

[16] Bahl M (2015). Architectural distortion on mammography: correlation with pathologic outcomes and predictors of malignancy. AJR Am J Roentgenol.

[17] Skaane P (2013). Comparison of digital mammography alone and digital mammography plus tomosynthesis in a population-based screening program. Radiology.

[18] Friedewald SM (2014). Breast cancer screening using tomosynthesis in combination with digital mammography. JAMA.

[19] Choudhery S (2021). Malignant outcomes of architectural distortion on tomosynthesis: a systematic review and meta-analysis. AJR Am J Roentgenol.

[20] Vijapura C (2018). Imaging features of nonmalignant and malignant architectural distortion detected by tomosynthesis. AJR Am J Roentgenol.

[21] Bahl M, Lamb LR, Lehman CD (2017). Pathologic outcomes of architectural distortion on digital 2D versus tomosynthesis mammography. AJR Am J Roentgenol.

[22] Ahmed SA (2022). Architectural distortion outcome: digital breast tomosynthesis-detected versus digital mammography-detected. Radiol Med.

[23] Vijayaraghavan GR, Newburg A, Vedantham S (2019). Positive predictive value of tomosynthesis-guided biopsies of architectural distortions seen on digital breast tomosynthesis and without an ultrasound correlate. J Clin Imaging Sci.

[24] Pujara AC, Hui J, Wang LC (2019). Architectural distortion in the era of digital breast tomosynthesis: outcomes and implications for management. Clin Imaging.

[25] Amitai Y (2020). Can breast MRI accurately exclude malignancy in mammographic architectural distortion?. Eur Radiol.

[26] Viala J (2013). Stereotactic vacuum-assisted biopsies on a digital breast 3D-tomosynthesis system. Breast J.

[27] Bohan S (2021). Diagnostic accuracy of tomosynthesis-guided vacuum assisted breast biopsy of ultrasound occult lesions. Sci Rep.

[28] Skaane P (2014). Two-view digital breast tomosynthesis screening with synthetically reconstructed projection images: comparison with digital breast tomosynthesis with full-field digital mammographic images. Radiology.

[29] Svahn TM (2015). Review of radiation dose estimates in digital breast tomosynthesis relative to those in two-view full-field digital mammography. Breast.

[30] Borg M, Badr I, Royle GJ (2013). A study to determine the differences between the displayed dose values for two full-field digitalmammography units and values calculated using a range of Monte-Carlo-based techniques: a phantom study. Radiat Prot Dosimetry.

[31] Suleiman ME, Brennan PC, McEntee MF (2017). Mean glandular dose in digital mammography: a dose calculation method comparison. J Med Imaging.

[32] Shin SU (2015). Comparative evaluation of average glandular dose and breast cancer detection between single-view digital breast tomosynthesis (DBT) plus single-view digital mammography (DM) and two-view DM: correlation with breast thickness and density. Eur Radiol.

[33] Feng SS, Sechopoulos I (2012). Clinical digital breast tomosynthesis system: dosimetric characterization. Radiology.

